# Genomic evidence for aerotrophy as a defining trait of Ktedonobacteria inhabiting silica-rich oligotrophic caves

**DOI:** 10.1093/ismeco/ycag175

**Published:** 2026-06-22

**Authors:** Andrea Firrincieli, Giacomo Broglia, Giulia Rizzo, Nicolas Greggio, Daniele Ghezzi, Bernardo Barosa, Francesco Sauro, Martina Cappelletti

**Affiliations:** Department for Innovation in Biological, Agro-Food and Forest Systems (DIBAF), University of Tuscia, 01100 Viterbo, Italy; Department of Pharmacy and Biotechnology (FaBit), University of Bologna, 40129 Bologna, Italy; Department of Pharmacy and Biotechnology (FaBit), University of Bologna, 40129 Bologna, Italy; Department of Biological, Geological and Environmental Sciences (BiGeA), University of Bologna, 48100 Ravenna Campus, Italy; Department of Pharmacy and Biotechnology (FaBit), University of Bologna, 40129 Bologna, Italy; Department of Pharmacy and Biotechnology (FaBit), University of Bologna, 40129 Bologna, Italy; La Venta Geographic Exploration Association, 31100 Treviso, Italy; Department of Geosciences, University of Padova, 35131 Padova, Italy; Department of Pharmacy and Biotechnology (FaBit), University of Bologna, 40129 Bologna, Italy; La Venta Geographic Exploration Association, 31100 Treviso, Italy

**Keywords:** oligotrophy, RuBisCO, CO_2_ fixation, Calvin–Benson–Bassham transaldolase variant, *Ktedonobacteraceae*, subterranean

## Abstract

Members of the class Ktedonobacteria (phylum Chloroflexota) are widespread across various terrestrial environments, including oligotrophic caves, although the genomic basis underlying this distribution remains unclear. Here, we present a systematic genomic analysis of Ktedonobacteria across ecosystems, with a focus on oligotrophic caves. Cave Ktedonobacteria belong to novel genera within the *Ktedonobacteraceae* and harbour genes associated with a mixotrophic metabolism combining the use of organic and inorganic substrates as energy and carbon sources. Comparative analyses of all available Ktedonobacteria genomes from diverse environments showed that most metabolic traits, including those associated with atmospheric gas oxidation, are primarily conserved among members of the same family. In contrast, genes involved in CO₂ fixation are enriched in Ktedonobacteria inhabiting caves. Phylogenetic analysis indicated that the RuBisCO of *Ktedonobacteria* likely represents a novel Form I subtype (named group IG) encompassing thermophilic and acidophilic bacteria from six different phyla that often inhabit similar extreme environments. The presence of this subtype across distinct lineages in comparable habitats suggests that it may confer a selective advantage in nutrient-poor settings (like caves) and that its distribution may be influenced by horizontal gene transfer. This inferred autotrophic capacity is associated with a transaldolase variant of the Calvin–Benson–Bassham cycle that was previously described only in a single Firmicutes species. Overall, this study provides genetic evidence for the potential coupling of atmospheric gas oxidation with dark CO₂ fixation in *Ktedonobacteria*, highlighting their possible role in sustaining primary production in oligotrophic ecosystems, including caves.

## Introduction

Caves are subterranean environments that support rich and diverse microbial communities, despite being generally characterized by oligotrophic conditions (i.e. low nutrient availability) due to the sporadic input of organic compounds from the surface and the absence of sunlight-driven photosynthesis [1–3]. Cave microorganisms have evolved metabolic strategies based on chemolithotrophic processes and the ability to establish synergistic interactions involving metabolite exchange [4, 5]. Among these strategies, cave microbes can scavenge nutrients released through rock weathering and from the oxidation of atmospheric trace gases (H₂, CO, and CH₄) [6, 7]. A metagenomic study further revealed an overrepresentation of RuBisCO genes in a speleothem sample from a carbonate cave compared with metagenomes from soil, rhizosphere, and marine environments, suggesting that CO₂ fixation in caves may occur through metabolic strategies independent of sunlight-derived energy [8].

Quartzite caves of the Guiana Shield tepuis are among the oldest cave systems ever discovered and studied, offering exceptional natural laboratories to investigate microbial colonization and rock–microbe interactions under aphotic and oligotrophic conditions. These ancient and remote subterranean systems are characterized by stable temperature and humidity, slight acidity due to the limited buffering capacity of quartzite, and very low amounts of organic nutrients [9, 10]. Previous studies have revealed complex and diverse microbial communities whose composition is strongly influenced by silica dissolution and precipitation, as well as water availability [9, 11, 12]. Microbiological analyses further indicated that members of the class *Ktedonobacteria*, unclassified at lower taxonomy levels, dominate biofilms that grow on quartzite rock in the deep parts of the caves [11, 13, 14].

Ktedonobacteria (phylum *Chloroflexota*) are mostly filamentous, aerobic bacteria with heterotrophic capacities, which have been predominantly characterized from different kinds of terrestrial environments [15], including nutrient-poor and extreme habitats, such as volcanic soils, geothermal fields, deserts, and silica-rich caves. In these environments, Ktedonobacteria appear to play an important role as pioneering microorganisms, possessing metabolic traits that enable survival under severe nutrient limitation and, in the case of volcanic soils, allow them to act as early colonizers during primary succession [16, 17]. Members of Ktedonobacteria have been reported from targeted gene analyses in the quartzite caves Imawarì Yeutà and Monte Cristo in South America and in the fumarolic ice cave Warren Cave in Antarctica [9, 13, 18, 19]. In these silica-rich cave systems, Ktedonobacteria were shown to harbour a large fraction of the genetic diversity associated with atmospheric trace gas oxidation and CO₂ fixation [18, 19]. They have also been reported to be abundant in microbial colonies and biofilms on quartzite rock, representing evidence of microbial growth and early quartzite alteration in deep cave environments [9, 13].

In this work, we present a systematic genomic investigation of *Ktedonobacteria* thriving in diverse ecological niches, including aphotic oligotrophic subterranean environments (caves). By classifying genomes according to their ecosystem of origin and performing comparative analyses, we identified genetic traits linked to cave colonization, providing insights into the metabolic strategies that may support the colonization and persistence of *Ktedonobacteria* in silica-rich oligotrophic subterranean ecosystems.

## Materials and methods

### DNA extraction and shotgun metagenomic sequencing

Total microbial DNA extraction from samples Ay304, Ay317, and Ay302 collected from Imawarì Yeutà Cave system included an initial pre-treatment consisting of incubating the samples for 30 min at 37°C with proteinase K (5 mg/ml) and lysozyme (8 mg/ml), followed by a lysis step with sodium dodecyl sulfate (0.1%) [20]. Then, DNA was extracted using the PowerLyzer PowerSoil Kit (Qiagen) according to the manufacturer’s protocol. Fifty ng of DNA was used for sequencing library preparation using the NEBNext Ultra™ II FS DNA Kit (New England Biolabs). The library was sequenced on a NextSeq 500 (Illumina) with the following specifications: read length 150 bp, paired-end, 7.5 Gb per sample. The DNA obtained from Ay302 and Ay317 samples was also sequenced using Oxford Nanopore Technology (ONT) with the SQK-LSK110 kit. The libraries were loaded on a FLO-MIN106D flow cell (chemistry R9.4.1).

### Metagenome assembly and reconstruction of metagenome-assembled genomes

For the reconstruction of metagenome-assembled genomes (MAGs) from Illumina sequencing data obtained from Warren Cave (raw sequencing data under the accession number PRJNA255918) and the Ay304 sample, raw reads were subjected to adapter removal and quality trimming using bbduk (https://sourceforge.net/projects/bbmap/), and finally assembled with MEGAHIT [21]. Contigs longer than 2.5 Kbp were binned using the metaWRAP “binning” and “binning_refinement” modules with default parameters [22]. The resulting bins with completeness >50% and contamination <10% were taxonomically classified using the Genome Taxonomy Database (GTDB, http://gtdb.ecogenomic.org) (GTDB-Tk) v.2.4.1 [23]. The quality of MAGs classified as Ktedonobacteria was further assessed using BUSCO v5.4.4 [24].

In the case of Ay317 and Ay302 metagenomes (sequenced via both Illumina and ONT), the ONT “*fast5”* signal data were first basecalled with gpu-guppy v6.2.0 using the super-accurate basecalling configuration (dna_r9.4.1_450bps_sup.cfg) with the following parameters enabled: “*--detect_adapter*,” “*--detect_mid_strand_adapter*,” and “*--trim_adapters*.” High-quality Illumina reads were processed as described above. High-quality ONT and Illumina reads were subsequently used as input for MetaPlatanus [25]. The scaffolds obtained after the gap-closing and polishing steps with TGS-GapCloser and NextPolish, respectively, were binned using the OPERA-MS [26] binning module “*OPERA-MS-UTILS.py binning*” and finally refined with the “binning_refinement” module of metaWRAP. The resulting bins were evaluated for completeness and contamination with BUSCO v5.4.4 and classified using GTDB-Tk v2.4.1.

### Functional annotation of oligotrophic cave Ktedonobacteria

Functional annotation and metabolic potential prediction of Ktedonobacteria MAGs recovered from Imawarì Yeutà, Warren Cave, and Monte Cristo Cave (MAGs retrieved from Figshare under DOI:10.6084/m9.figshare.15106227) were performed using anvi’o v7.1 [27]. First, FASTA files for MAGs were converted into anvi’o contigs databases with the program “anvi-gen-contigs-database,” which uses Prodigal v2.6.3 [28] to identify open reading frames on contigs. The resulting genes were then annotated against the PFAM database [29] and the orthologous gene families (KOfams) database [30] by the Kyoto Encyclopedia of Genes and Genomes (KEGG) [31] using the programs “anvi-run-pfams” and “anvi-run-kegg-kofams” with default parameters [32]. In addition, dbCAN v3 was used to predict the carbohydrate degradation potential [33].

### Construction of the Ktedonobacteria genome dataset and ecological niche classification

A dataset comprising a total of 111 Ktedonobacteria genomes was constructed by integrating 8 MAGs reconstructed from oligotrophic silica-rich cave environments with 103 publicly available Ktedonobacteria genomes retrieved from the NCBI GenBank database (accessed in February 2023; Table S1). We grouped these genomes into six categories based on their ecological niche: silica-rich oligotrophic caves (8 genomes), vegetated soil/rhizosphere (35 genomes), geothermal soil/volcanic area (18 genomes), permafrost/Antarctic soil (12 genomes), acid mine drainage/polluted soil (23 genomes), and oligotrophic soil/rock (7 genomes). In addition, 8 genomes from unknown environments or habitats that could not be categorized were included in the dataset (defined as “unknown” in the figures and tables).

The vegetated soil/rhizosphere category includes genomes from environments like meadow soil, black locust and pine trees, and fertilized topsoil. The category oligotrophic soil/rock differs from vegetated soil/rhizosphere by its lower nutrient availability and includes MAGs reconstructed from metagenomic data of a subsurface holobiont of the plant *Barbacenia macrantha,* which colonizes nutrient-poor quartzite rocky outcrops in Brazil [34]. The geothermal soil/volcanic area category is characterized by high temperatures, including genomes recovered from thermal soils located in volcanic areas and underground burning abandoned coal mines. The permafrost/Antarctic soil category comprises MAGs obtained from tundra permafrost regions and polar desert biomes sampled at different depths. These regions are characterized by extreme aridity, low temperatures, and are classified as mineral soils with high contents of calcium, magnesium, potassium, and sodium [35]. The acid mine drainage/polluted soil category includes genomes retrieved from sediments and soils associated with acid mine drainages and polluted sites.

### Phylogenomic and pangenomic analyses of Ktedonobacteria

A phylogenomic tree including all 111 Ktedonobacteria genomes in the dataset was generated using the GTDB-Tk de novo workflow. The concatenated alignment of 120 single-copy bacterial marker genes (bac120) was used as input for IQTREE v2.2.5 to compute a maximum-likelihood phylogeny using the WAG substitution model and ultrafast bootstrap (1000 replicates). The resulting tree was visualized and annotated using iTOL.

The pangenomic analysis of the 111 Ktedonobacteria genomes was performed using the pangenomics workflow implemented in anvi’o v7.1 [36]. This analysis combined genomes from this study with those recovered from the NCBI GenBank database (Table S1). The Ktedonobacteria genomes from the NCBI were first turned into anvi’o contigs databases and annotated using “anvi-run-pfams” and “anvi-run-kegg-kofams” with default parameters. The pangenome was then computed using the program “anvi-pan-genome” with the “mcl-inflation” parameter set to 2 and “minbit” set to 0.5, to identify clusters of ortholog genes between distantly related genomes.

### Phylogenetic and synteny analyses of RuBisCO genes and gene clusters in Ktedonobacteria

Non-redundant (90% cluster identity) RuBisCO large subunit (*rbcL*) sequences, representing different subtypes of the Form I, were retrieved from Prywes *et al.* [37]. Sequences were aligned with MAFFT v7.310 [38] using the settings –globalpair (G-INS-i algorithm), --maxiterate (1000 iterations) and --reorder. Poorly aligned regions were trimmed with trimAl using a gap threshold (-gt) of 0.1 [39]. The alignment file was analysed in IQTREE v2.2.5 [40] to build a maximum-likelihood phylogenetic tree using ModelFinder Plus (-m MFP), the -bnni optimization, and ultrafast bootstrap (-bb 1000). The resulting consensus tree was constructed under the Q.pfam+R10 substitution model and visualized with iTOL.

Synteny analysis of RuBisCO gene clusters in Ktedonobacteria was performed using clinker with default parameters (https://github.com/gamcil/clinker). Briefly, contigs possessing one copy each of the RuBisCO subunits were reannotated using prokka v1.14.6 [41]. The resulting GenBank files were finally aligned with clinker to visualize gene cluster organization.

To verify the absence of sedoheptulose-1,7-bisphosphatase (SBPase) or bifunctional FBPase/SBPase, a targeted tBLASTN search was performed on MEGAHIT-assembled metagenomes (--presets meta-large --min-contig-len 1500) containing RuBisCO-positive Ktedonobacteria MAGs. A non-curated set of SBPase sequences was downloaded from UniProtKB (March 2026), targeting Bacteria annotation records containing the terms “sedoheptulose-bisphosphatase” or “sedoheptulose-1,7-bisphosphatase.” Contigs producing significant hits (minimum identity 40% and e-value ≤1e^−20^) were taxonomically classified against the NCBI non-redundant protein database (March 2026) [42] using MMseqs2 taxonomy (v18-8cc5c) with the following settings enabled: “--tax-lineage 1,” “--lca-ranks superkingdom,phylum,class,order,family,genus,species,” “--majority 0.5,” “--vote-mode 1,” and “--orf-filter 1” [43].

### [NiFe]-hydrogenase classification

To classify the [NiFe]-hydrogenases detected in the Ktedonobacteria pangenome, all sequences included in gene clusters GC_00002278, GC_00014573, and GC_00016658, which contain proteins annotated as a hydrogenase large subunit (K06281), were aligned against the HydDB database [44] using DIAMOND-BLASTP [45], following the protocol described at https://github.com/GreeningLab/HydDB.

### Statistical analyses

Jaccard distances were computed from *Ktedonobacteria* KEGG Ortholog (KO) presence/absence matrices generated with anvi’o, using the microViz R package v0.12.4. A PERMANOVA with 999 permutations was then conducted to assess differences in KO composition among Ktedonobacteria families. The identification of family- and cave-specific KO was performed using the Rao’s Score test via “anvi-compute-functional-enrichment-in-pan” [46]. The analysis was performed to identify both family-specific and cave-specific functional genes using an adjusted *q*-value <0.01. For the differential distribution of specific hydrogenase subgroups, the enrichment analysis via “anvi-compute-functional-enrichment-in-pan” was performed using gene cluster IDs as functions rather than KO.

To assess the robustness of these results to the genome quality filtering criteria defined via BUSCO, the enrichment analysis was repeated by excluding 16 genomes showing contamination >10% as estimated by CheckM2 [47]. Cave-specific KOs that were significantly enriched (adjusted *q*-value <0.01 in both full and restricted sets) were further validated via HAPPI [48], which models gene detection probability against BUSCO completeness and conducts a sensitivity analysis at ε = 0.10 to account for spurious detections from contamination. The resulting *P*-values were corrected using the Benjamini–Yekutieli (BY) procedure [49].

### Total organic carbon quantification

CHNS elemental analysis was performed after cave sample drying, homogenization, and weighing of ~10 mg aliquots. Organic carbon was determined following double HCl pre-treatment, with intermediate oven drying, to remove the inorganic (carbonate) fraction. Organic carbon was calculated as the difference between total and inorganic carbon and normalized to sample weight.

Analytical accuracy was assessed using a certified reference material (*N* = 0.2100%, *C* = 2.2900%) analysed under identical conditions.

## Results

### Reconstruction of Ktedonobacteria genomes from caves

MAGs of Ktedonobacteria inhabiting cave environments were reconstructed from sequencing data from samples collected in the Imawarì Yeutà cave (Auyán Tepui, Venezuela), Monte Cristo Cave (Diamantina, Brazil), and Warren Cave (Mt. Erebus, Antarctica). These cave systems were characterized by a high amount of silica in the rock substrate, slightly acidic pH, and a very low amount of organic carbon, with values below 0.1% indicating extremely oligotrophic conditions (Table S2).

Four MAGs affiliated with the class Ktedonobacteria were assembled from Imawarì Yeutà cave samples, i.e. one from the sample Ay304 and three from Ay302 (Table S1). Sample Ay317 did not yield any Ktedonobacteria MAGs of sufficient quality. Two MAGs affiliated with Ktedonobacteria were reconstructed from Warren Cave samples (Table S1). Together with the previously reconstructed MAGs from the Monte Cristo Cave [18], these genomes comprise the dataset of eight Ktedonobacteria MAGs inhabiting oligotrophic caves, which were analysed for their metabolic potential.

### Metabolic potential of cave Ktedonobacteria

#### Central carbon metabolism and carbon fixation

**Figure 1:**
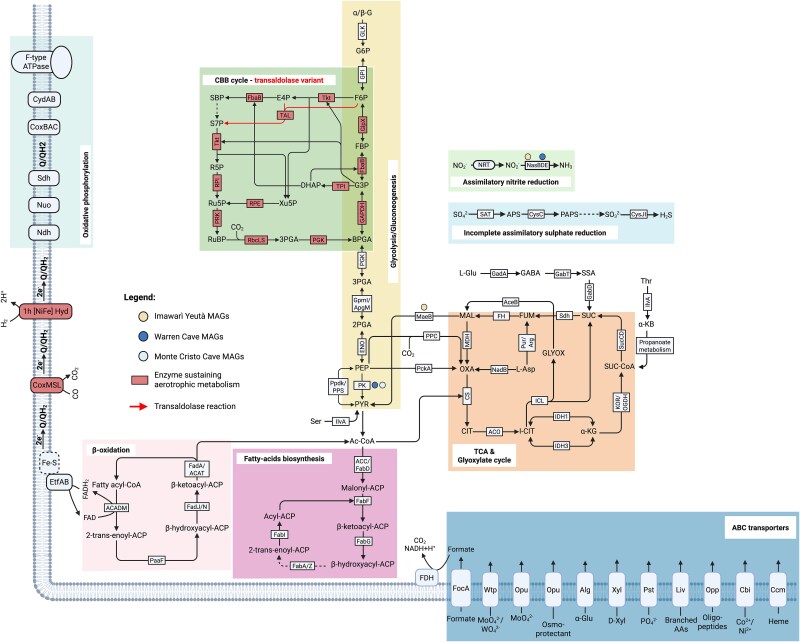
Genome-based metabolic reconstruction of Ktedonobacteria inhabiting silica-rich oligotrophic caves. A summary of the functional annotation for all cave Ktedonobacteria MAGs used to generate this figure is provided in Table S3. Dashed lines indicate enzymes/reactions missing in all cave Ktedonobacteria. Enzymes/reactions sustaining aerotrophic metabolism are shown in red. Coloured dots highlight enzymes/reactions detected only in specific cave Ktedonobacteria. Abbreviations of metabolites: α/β-G, α/β-glucose; G6P, glucose-6-phosphate; PEP, phosphoenolpyruvate; PYR, pyruvate; Ac-CoA, acetyl CoA; Ser, serine; OXA, oxaloacetate; CIT, citrate; I-CIT, isocitrate; α-KG, α-ketoglutarate; SUC-CoA; succinyl-CoA; SUC, succinate; GLYOX, glyoxylate; FUM, fumarate; MAL, malate; L-Asp, L-aspartate; α-KB, α-ketobutyrate; L-Glu, L-glutamate; SSA, succinate semialdehyde; D-Xyl, D-xylose; GABA, γ-aminobutyric acid; RuBP, ribulose-1,5-bisphosphate; 3PGA, 3-phosphoglycerate; BPGA, 1,3-bisphosphoglycerate; DHAP, dihydroxyacetone phosphate; G3P, glyceraldehyde-3-phosphate; FBP, fructose-1,6-bisphosphate; F6P, fructose-6-phosphate; E4P, erythrose-4-phosphate; SBP, sedoheptulose-1,7-bisphosphate; S7P, sedoheptulose-7-phosphate; R5P, ribose-5-phosphate; Ru5P, ribulose-5-phosphate.

Ktedonobacteria MAGs from oligotrophic caves possessed genes coding for enzymes of complete/nearly complete pathways of glycolysis/gluconeogenesis, pentose phosphate pathway, tricarboxylic (TCA) cycle, synthesis and β-oxidation of fatty acids, and CO_2_ fixation through the Calvin–Benson–Bassham (CBB) cycle (Fig. 1).

Analysis of the glycolytic pathway indicated that cave Ktedonobacteria MAGs in this study possessed the genes involved in the Embden–Meyerhof–Parnas variant of glycolysis. However, MAGs from Imawarì Yeutà lacked the pyruvate kinase (*pk*, K00873) gene, an absence apparently compensated by the presence of the PPi-dependent pyruvate, orthophosphate dikinase (*ppdk*, K01006) and pyruvate, water dikinase (*pps*, K01007) genes, both of which catalyse the reversible conversion of phosphoenolpyruvate (PEP) to pyruvate (Fig. 1). Cave Ktedonobacteria included in this study also possessed a complete set of genes involved in the oxidative (module M00006) and non-oxidative (module M00007) phases of the pentose phosphate pathway, allowing the generation of precursors, for the biosynthesis of nucleotides and amino acids (Table S3).

A complete set of genes encoding the enzymes of the TCA cycle was detected, together with additional genes catalysing anaplerotic reactions that replenish TCA intermediates (Fig. 1). Specifically, cave Ktedonobacteria under analysis possessed (i) phosphoenolpyruvate carboxylase gene (*ppc*, K01595), which catalyses the addition of CO₂ to phosphoenolpyruvate to form oxaloacetate, (ii) the genes encoding isocitrate lyase (*icl,* K01637) and malate synthase (*aceB*, K01638), two key enzymes of the glyoxylate cycle, which enable the net conversion of two acetyl-CoA molecules into succinate. The resulting succinate can subsequently enter the TCA cycle or be used for amino acid biosynthesis. Other genes indicated the capability of cave Ktedonobacteria included in this study to use amino acids as carbon sources through the synthesis of various TCA intermediates. These included the formation of (i) fumarate via the activity of enzymes encoded by *purAB* (K01939-K01756) and *argGHA* (K01940-K01755-K01468), (ii) oxaloacetate via aspartate deamination (*nadB*, K00278), (iii) succinate via glutamate conversion (*gadA*, K01580; *gabT*, K07250; *gabD*, K00135); and (iv) succinyl-CoA via threonine deamination in the propanoyl-CoA metabolism (*ilvA*, K01754 in module M00741).

With respect to carbon fixation, genes encoding the key enzymes of the 3-hydroxypropionate bicycle and the Wood-Ljungdahl pathway, which are typically present in anaerobic and facultative aerobic members of Chloroflexota performing light-driven photosynthesis, were absent [50]. Conversely, cave Ktedonobacteria possessed genes encoding the large (*rbcL*, K01601) and small (*rbcS*, K01602) subunits of ribulose bisphosphate carboxylase/oxygenase (RuBisCO), phosphoribulokinase (*prk*, K00855), and the gene encoding transaldolase/fructose-6-phosphate aldolase (TAL, PF00923) (Fig. 1). The latter enzyme is involved in the transaldolase variant of the CBB cycle, in which the transaldolase reaction regenerates ribulose-1,5-bisphosphate (RuBP), the CO₂ acceptor in the cycle [51].

#### Energy metabolism

The electron transport chain of the cave Ktedonobacteria included the main components of the respiratory chain including those for the non-electrogenic NADH:quinone reductase (*ndh*, K03885), NADH:ubiquinone oxidoreductase (*nuo,* module M00144) complex, succinate dehydrogenase (*sdh,* module M00149), cytochrome bd complex (*cydAB,* module M00153), cytochrome bcc:aa3 complex (*coxBAC,* module M00155), and F-type ATP synthase (module M00157) (Fig. 1). Additional genes encoded complexes able to transfer electrons either directly to the quinone pool of the respiratory chain or indirectly via reducing equivalents (NADH and FADH_2_) generated through the Nuo complex. The presence of genes coding for the electron transfer flavoproteins (*etf*, K03522-K03521-K00313) and formate dehydrogenase (*fdh*, K00124) suggested that these bacteria couple the oxidation of fatty acids (β-oxidation) and formate with energy metabolism through the production of FADH_2_ and NADH, respectively. Likewise, the presence of genes encoding the aerobic carbon monoxide dehydrogenase complex (*coxMSL,* K03519-K03518-K03520), and the high-affinity, oxygen-insensitive Group 1h [NiFe] hydrogenase (Table S4) complex indicated that the cave Ktedonobacteria had the genetic potential to generate a proton gradient for ATP synthesis through atmospheric trace gase oxidation.

#### Sulphur and nitrogen metabolism

Enzymes involved in the dissimilatory sulphur and nitrogen metabolism were absent, suggesting that they were unable to couple the reduction of inorganic nitrogen and sulphur compounds with energy metabolism (Fig. 1). Similarly, cave Ktedonobacteria appeared to have a limited capacity to assimilate nitrogen and sulphur in the form of nitrate and sulphate, as they lacked the genes encoding the enzymes that catalyse the first reduction step of these compounds into nitrite and sulphite, respectively (Fig. 1). Conversely, genes encoding the sulphite reductase (NADPH) flavoprotein (*cys*) and the nitrite reductase [NAD(P)H] (*nas*) were detected. They catalyse the final reactions of the nitrate and sulphate assimilatory pathways, converting nitrite and sulphite into ammonia and sulphide, respectively.

#### ABC transporters and carbohydrate-active enzymes

Cave Ktedonobacteria under analysis possessed genes encoding several ABC transporters involved in uptake of (i) mineral ions, such as tungstate/molybdate (*wptABC*), nickel/cobalt (*cbiNMQO*), iron in the form of heme (*ccmABCD*), and phosphate (*pstABCS*); (ii) branched-chain amino acids (*livKMHGF*) and peptides (*oppABCDF*); (iii) mono- and oligosaccharides, like alpha-glucosides (*algEFGK*), trehalose/maltose (*thuEGFK*), rhamnose (*rhaSPQT*), and ribose/D-xylose (*rbsBCAD*) (Fig. 1). With respect to the uptake of mono- and oligosaccharides, various genes encoding carbohydrate-active enzymes (CAZy), which catalyse the hydrolysis of di- and oligosaccharides deriving from complex polysaccharides such as chitin, cellulose, and hemicellulose (Table S5), were detected. However, CAZy with a signal peptide were only detected in a few MAGs from Warren Cave and Imawarì Yeutà, suggesting that most polysaccharide-degrading enzymes were likely intracellular. This pattern suggested that cave Ktedonobacteria included in this study were not primary degraders of complex polysaccharides but instead scavenged soluble oligosaccharides released by other polysaccharide-degrading microorganisms.

### Cave Ktedonobacteria belong to novel genera of the Ktedonobacteraceae family

**Figure 2:**
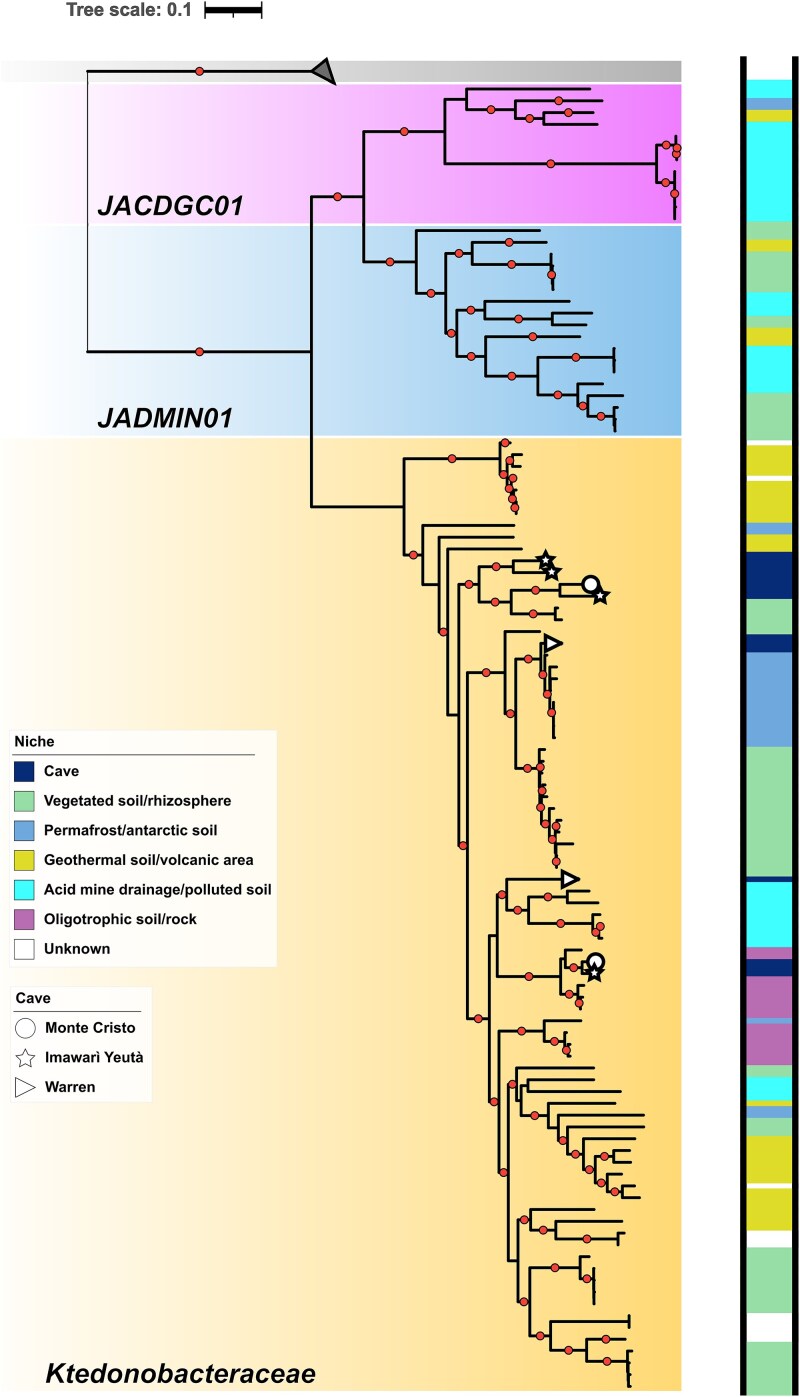
The maximum-likelihood phylogenomic tree illustrates the affiliation of the cave MAGs with the Ktedonobacteria families JACDGC01, JADMIN01, and *Ktedonobacteraceae*. Colour strips highlight the environmental niche of each genome: silica-rich oligotrophic cave, vegetated soil/rhizosphere, permafrost/Antarctic soil, geothermal soil/volcanic area, acid mine drainage/polluted soil, oligotrophic soils/rocks, and uncharacterized habitats. Cave-specific symbols at the branch tips indicate MAGs from Monte Cristo Cave (circle), Imawarì Yeutà Cave (star), and Warren Cave (triangle). Bootstrap values >80% are indicated by red dots. Collapsed clades correspond to the *Dehalococcoides mccartyi* genomes GCF_000011905.1, GCF_001889305.1, and GCF_000830885.1 that were used as outgroups (coloured in grey).

The phylogenomic analysis comprised all 111 Ktedonobacteria genomes in the dataset, including 8 MAGs from oligotrophic caves and 103 genomes retrieved from the NCBI GenBank database (accessed in February 2023; Table S1). The phylogenomic tree showed that Ktedonobacteria formed a monophyletic class, divided into three families, i.e. *Ktedonobacteraceae*, JACDGC01, and JADMIN01 (Fig. 2). In contrast with the uncharacterized families JACDGC01 and JADMIN01, which almost exclusively included MAGs from acid mine drainage/polluted soil and vegetated soil/rhizosphere, *Ktedonobacteraceae* genomes were found across all niches defined in this study, including silica-rich oligotrophic caves. Within *Ktedonobacteraceae*, the Imawarì Yeutà MAGs clustered with other MAGs from caves and were assigned to yet uncharacterized genera (Table S1).

### Taxonomy affiliation is the main driver of functional diversity in Ktedonobacteria

**Figure 3:**
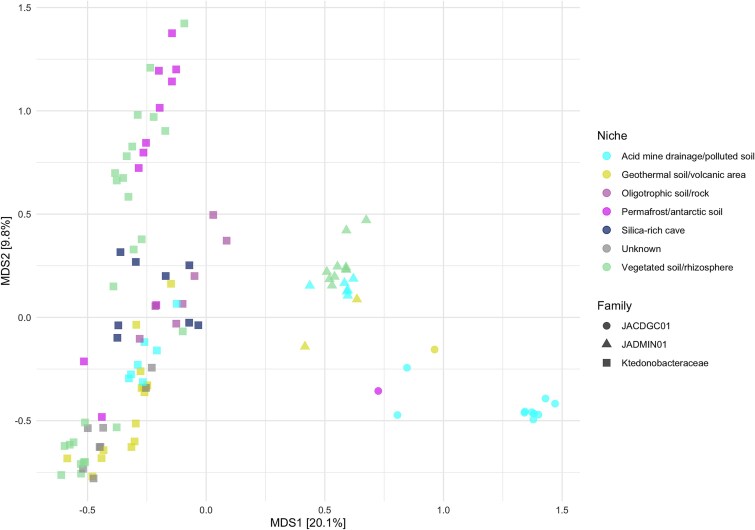
Principal coordinate analysis (PCoA) based on Jaccard distances computed on presence/absence profiles of KEGG orthologs across Ktedonobacteria genomes. Points are coloured according to the environmental niche of origin (silica-rich oligotrophic cave, acid mine drainage/polluted soil, geothermal/volcanic area, oligotrophic soil/rock, permafrost/Antarctic soil, vegetated soil/rhizosphere, or unknown). Point shapes indicate taxonomic affiliation, i.e. JACDGC01 (circle), JADMIN01 (triangle), and *Ktedonobacteraceae* (square). The percentage of variation explained by each axis is shown in brackets.

Comparative genomic analysis of all Ktedonobacteria genomes, based on the functional profiles of 3023 KOs, revealed that both niche (*R*^2^ = 0.15, *P* = .001) and family (R^2^ = 0.193, *P* = .001) significantly influenced functional variation across members of this class (Fig. 3). However, we observed a degree of confounding between niche and family among members of JADMIN01. Indeed, most representatives of this family were retrieved from acid mine drainage environments, making it difficult to distinguish the effects of ecological niche from those of taxonomy.

**Figure 4:**
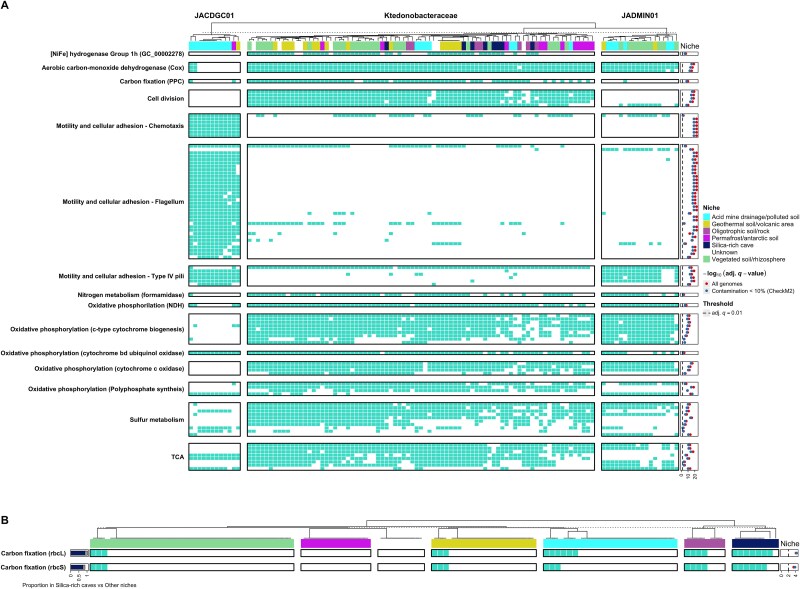
(A) Heatmap showing the presence/absence of family-specific KEGG orthologs significantly associated with Ktedonobacteria families JACDGC01, *Ktedonobacteraceae*, and JADMIN01 (differential enrichment analysis results are available in Tables S6.1, S6.2). Columns and rows are clustered based on taxonomic groups (JACDGC01, *Ktedonobacteraceae*, and JADMIN01) and functional modules, respectively. Columns are coloured according to the environmental niche. (B) Heatmap showing the presence/absence of cave-specific KEGG orthologs of *rbcL* and *rbcS* genes across Ktedonobacteria genomes from different environmental niches (differential enrichment analysis results are available in Table S6.3). Bars show the proportion of genomes containing each gene within each environmental niche. Adjusted *q*-values from “anvi-compute-functional-enrichment-in-pan” obtained from different Ktedonobacteria genome datasets defined according to BUSCO (red dots) and CheckM2 (blue dots) genome quality filtering criteria are shown as a dot plot.

A total of 1011 KOs were family-specific (adjusted *q*-value <0.01; Table S6.1), consistently across both the full and reduced datasets excluding 16 genomes with contamination >10% (CheckM2). Among these, 95 KO terms were predominantly found in one specific family (prevalence >0.80 in these individuals and prevalence <0.50 in the others) (Fig. 4a). The most marked differences associated with taxonomic lineage were observed in genes encoding proteins of the cell division machinery, flagellum and chemotaxis, and Type IV pili. Notably, genes of the cell division machinery were detected exclusively in *Ktedonobacteraceae*, whereas flagellar and chemotaxis genes were enriched in JACDGC01. Likewise, Type IV pili proteins were broadly present in JADMIN01 and JACDGC01 but largely absent in *Ktedonobacteraceae*. Other family-specific KOs were genes involved in the TCA cycle, oxidative phosphorylation, nitrogen and sulphur metabolism, carbon fixation (PPC), and atmospheric trace gas oxidation (hydrogenases and carbon monoxide dehydrogenases) (Fig. 4a). Specifically, aerobic carbon monoxide dehydrogenase genes (*cox* genes) were significantly associated with the families JADMIN01 and *Ktedonobacteraceae,* while Group 1h [NiFe] hydrogenase genes (gene cluster GC_00002278), which are involved in high-affinity H₂ oxidation at atmospheric concentrations, were significantly overrepresented only in *Ktedonobacteraceae* (Table S6.2).

### RuBisCO genes are significantly enriched in the genomes of Ktedonobacteria inhabiting oligotrophic caves

The association between the isolation source and functional potential was also examined. Considering those functions detected as cave-specific (Table S6.3), which remained robust in a sensitivity analysis, six KOs were significantly enriched in Ktedonobacteria inhabiting oligotrophic silica-rich caves (Table S6.4). These functions were related to toxin–antitoxin systems and lipid metabolism (e.g. soluble epoxide hydrolase). Moreover, key genes of the CBB cycle, such as *rbcL* and *rbcS*, were also significantly enriched (Fig. 4b) and detected in at least one MAG retrieved from each silica-rich oligotrophic cave. A few other non-cave MAGs harboured *rbcLS* genes, and they derived from oligotrophic soil/rock, geothermal soil/volcanic area, and acid mine drainage/polluted soils (Fig. 4b).

### Phylogenetic analysis of RuBisCO genes and CO_2_-fixation potential in Ktedonobacteria

#### Ktedonobacteria possess a novel Form I subtype of RuBisCO

**Figure 5:**
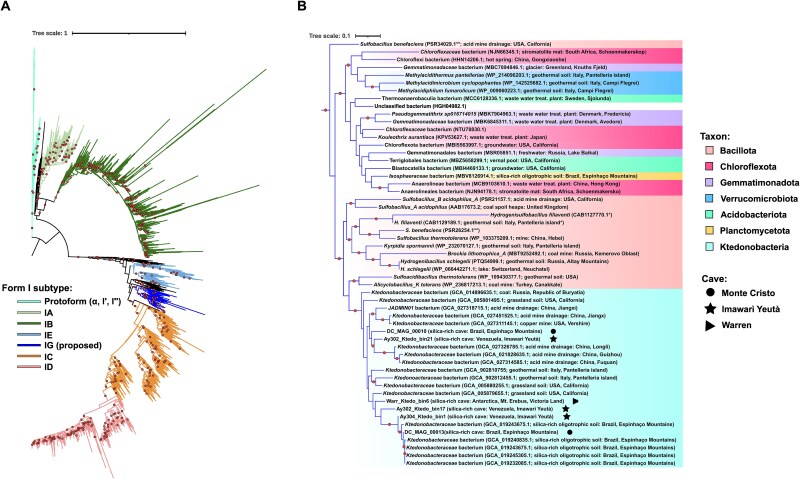
Phylogenetic analysis and environmental distribution of RuBisCO Form I large subunit (RbcL) from Ktedonobacteria. (A) Maximum-likelihood phylogenetic tree of known and unknown RbcL Form I subtypes highlighted with different colours according to Prywes *et al.* [37]. In blue, the proposed subtype IG. The interactive version of the tree shown in panel A is available at: https://itol.embl.de/shared/JPRm2DjUXxFP. (B) Expanded phylogeny showing the placement of Ktedonobacteria RbcL within Form IG with colours highlighting the taxon in which the RbcL was found. Bootstrap values >80% are indicated with red dots. Asterisks indicate RbcL genes detected in the same genome.

To better understand the origin of CO_2_ fixation via the CBB cycle in Ktedonobacteria, we built a phylogenetic tree encompassing 1328 RbcL Form I sequences representing the subtypes Iα, I′, I″, IA, IB, IC, ID, and IE (Fig. 5a)*.* All Ktedonobacteria RbcL Form I were included within a well-supported monophyletic clade (95% ultrafast bootstrap support) together with RbcL Form I from extremophilic and/or chemolithoautotrophic strains of Bacillota, non-Ktedonobacteria Chloroflexota, Gemmatimonadota, Verrucomicrobiota, Acidobacteriota, and Planctomycetota (Fig. 5b). This clade, hereafter referred to as IG, was phylogenetically distinct from subtype IE, which exhibited a more restricted taxonomic distribution (Actinomycetota). Conversely, IG spanned six phyla, indicating a broad phylogenetic distribution likely shaped by horizontal gene transfer events. Furthermore, organisms harbouring the IG were associated with similar ecological niches, such as geothermal soils, mines, and silica-rich oligotrophic environments. For instance, the thermoacidophilic facultative chemolithoautotrophic Bacillota [52–56], Verrucomicrobiota [57], and Ktedonobacteria colonizing geothermal soil, all harboured the IG subtype (Fig. 5b). Similarly, the IG subtype was detected in Ktedonobacteria and Planctomycetota from oligotrophic silica-rich soils in the Espinhaço Mountains region, in which the Monte Cristo cave is located.

#### Ktedonobacteria possess a Form I RuBisCO-mediated transaldolase variant of the CBB cycle

**Figure 6:**
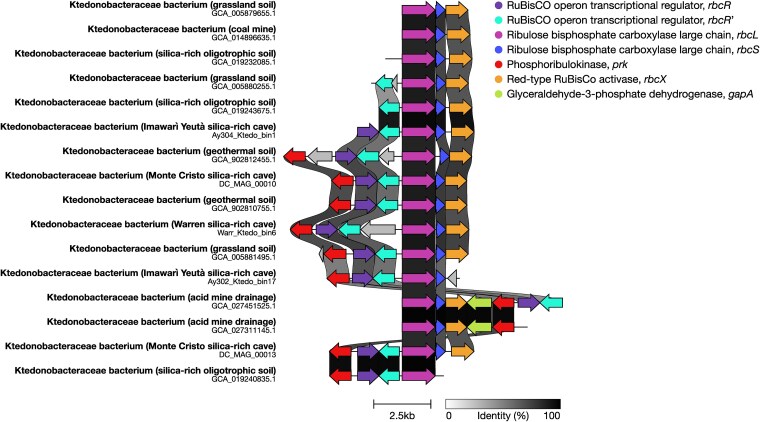
Conservation of the RuBisCO gene cluster and gene neighbourhood in cave and non-cave Ktedonobacteria. Protein-coding genes are coloured according to functional annotation. Conserved syntenic blocks are highlighted by gradient bars with darker shading representing higher similarity. Genomic neighbourhood comparisons are shown only for Ktedonobacteria genomes that contain a partially complete RuBisCO cluster (*rbcR–rbcL–rbcS*).

Ktedonobacteria were then analysed for the presence of genes associated with CO_2_ fixation via the CBB cycle. This analysis revealed that the *rbc* gene cluster was mostly conserved across Ktedonobacteria members, with some genes more conserved than others (Fig. 6). Apart from *rbcLS*, other conserved genes in the cluster encoded the LysR family transcriptional regulator (*rbcR* or *cbbR*, K21703) and the red-type RuBisCO activase (*rbcX*). The *cbbR* genes were always present in two copies and located upstream of the *rbcLS* genes. The *rbc* gene cluster also included genes encoding other CBB cycle enzymes, such as the glyceraldehyde-3-P dehydrogenase A (*gapA,* K00134) and phosphoribulokinase (*prk*, K00855).

**Figure 7:**
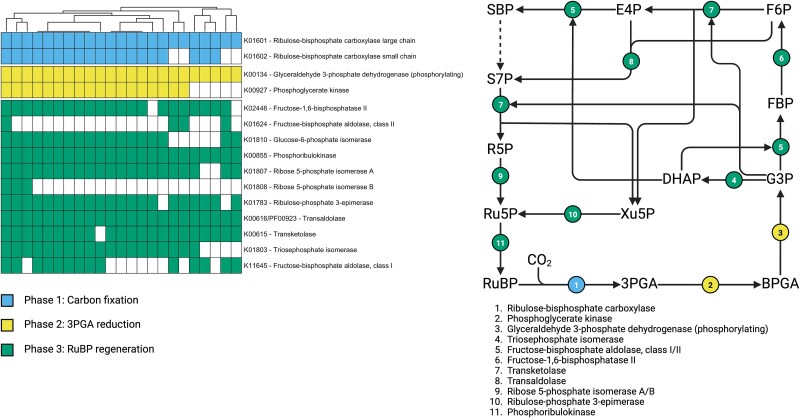
Heatmap showing the presence/absence of KEGG orthologs in Ktedonobacteria associated with the three phases of the CBB cycle, i.e. carbon fixation, 3-phosphoglycerate (3PGA) reduction, and ribulose-1,5-bisphosphate (RuBP) regeneration. Rows are clustered according to the phases of the CBB cycle (carbon fixation, 3-PGA reduction, and RuBP regeneration). In the schematic representation of the CBB cycle, coloured numbered circles correspond to the KO entries (1–11). Abbreviation: RuBP, ribulose-1,5-bisphosphate; 3PGA, 3-phosphoglycerate; BPGA, 1,3-bisphosphoglycerate; DHAP, dihydroxyacetone phosphate; G3P, glyceraldehyde-3-phosphate; FBP, fructose-1,6-bisphosphate; F6P, fructose-6-phosphate; E4P, erythrose-4-phosphate; SBP, sedoheptulose-1,7-bisphosphate; S7P, sedoheptulose-7-phosphate; R5P, ribose-5-phosphate; Ru5P, ribulose-5-phosphate.

As for the genes of the CBB cycle that are not co-localized within the *rbcLS* gene cluster, we found all the genes except those encoding sedoheptulose-1,7-bisphosphatase (SBPase) and the bifunctional fructose-1,6-bisphosphatase II/sedoheptulose-1,7-bisphosphatase (Fbp-SEBP; GlpX-SEBP). Instead, we found that all the Ktedonobacteria genomes containing at least one copy of either *rbcL* or *rbcS* harboured the transaldolase gene that allows the bypass of the SBPase reaction (Fig. 7).

No SBPase or bifunctional FBPase/SBPase enzymes were detected in any of the genomes under analysis and in Ktedonobacteria-classified contigs assembled from metagenomes in which Ktedonobacteria MAGs harbouring RuBisCO genes were detected. The resulting protein hits did not include any enzymes associated with SBPase activity (Table S7). Altogether, these findings support the hypothesis that CO₂ fixation in Ktedonobacteria likely proceeds through a non-canonical variant of the CBB cycle.

## Discussion

In this study, we explored the metabolic potential of members of the class Ktedonobacteria, a still poorly characterized taxon within the phylum Chloroflexota, which often emerges as key players in extreme habitats characterized by nutrient limitations, extreme pH conditions, and/or temperatures [15]. Among these habitats, Ktedonobacteria have been reported to be abundant in silica-rich oligotrophic caves, i.e. Imawarì Yeutà, Monte Cristo Cave, and Warren Cave [11, 13, 18, 19]. These caves share key features, including silica-rich substrates (>50% SiO₂), slightly acidic pH (pH of 4–5), strong organic nutrient limitation (<0.1% w/w), and elevated CO₂ concentrations (ranging from ~0.5% to 2% in Warren Cave) (Table S2) [58].

To investigate the genomic bases underlying the ability of Ktedonobacteria to colonize these subterranean environments, we first analysed their metabolic potential, using the *Ktedonobacteria* MAGs reconstructed in this study (from Imawarì Yeutà and Warren Cave) and those available from a previous study (from Monte Cristo Cave). The results indicated that the Ktedonobacteria inhabiting silica-rich oligotrophic caves possess genetic traits associated with a mixotrophic metabolism, suggesting the potential to utilize both organic substrates and inorganic CO₂ as carbon sources, while deriving energy from the oxidation of atmospheric trace gases or organic compounds. The capacity to switch between these metabolic modes may represent a key adaptive strategy in oligotrophic cave environments, where the availability of organic carbon is limited and sporadic.

The results from a large-scale comparative analysis of Ktedonobacteria showed that the functional diversity within this class was primarily associated with taxonomic lineage rather than with ecological niche. This pattern suggests that their metabolic traits are largely conserved within phylogenetic lineages, with limited influence from environmental adaptation. Similar trends have been reported for other bacterial groups with low rates of horizontal gene transfer, in which vertical inheritance of metabolic functions predominates over niche-specific gene acquisition [59].

All silica-rich oligotrophic cave MAGs belonged to novel genera of *Ktedonobacteraceae* and shared key functional traits with other members of this family. In particular, genetic functions associated with the oxidation of atmospheric trace gases, such as genes encoding oxygen-insensitive Group 1h [NiFe] hydrogenases and aerobic carbon monoxide dehydrogenases (Cox), were commonly found in members of the *Ktedonobacteraceae*. While *cox* genes were also detected in members of the JADMIN01 family, Group 1h [NiFe]-hydrogenase genes were absent in both JADMIN01 and JACDGC01. The presence of high-affinity hydrogenases in *Ktedonobacteraceae* may represent a key trait supporting their broader ecological distribution compared to the other two families. Indeed, the ability to oxidize H₂ at atmospheric concentrations enhances metabolic flexibility and provides a competitive advantage in nutrient-poor environments [60]. This metabolic versatility may compensate for the more limited repertoire of genes associated with motility and adhesion compared to JACDGC01, allowing *Ktedonobacteraceae* to colonize and persist in oligotrophic environments by relying on atmospheric trace gas oxidation for energy rather than on active motility for nutrient acquisition [61].

Although most genetic traits of *Ktedonobacteraceae* were conserved across members of the same family, a few functions were significantly associated with *Ktedonobacteria* inhabiting silica-rich oligotrophic caves. Among these, our results highlighted the enrichment of *rbcL* and *rbcS*, which are key genes for CO₂ fixation and primary production. In particular, *Ktedonobacteraceae* members inhabiting subterranean environments possessed specific genetic functions involved in CO__2__ fixation through a non-canonical pathway known as the RuBisCO-mediated transaldolase variant. This variant of the CBB cycle was only identified and characterized in *Thermodesulfobium acidophilus* (of the Firmicutes phylum) [51]. This chemolithoautotrophic bacterium performs autotrophic CO₂ fixation via the CBB pathway despite lacking the sedoheptulose-1,7-bisphosphatase (SBPase) enzyme, which is required for the regeneration of ribulose-1,5-bisphosphate (RuBP). The SBPase reaction is bypassed by the transaldolase (TAL) enzyme, which functionally replaces SBPase and regenerates RuBP through an alternative route [51]. Notably, unlike *Thermodesulfobium acidophilus,* which possesses a Form III RuBisCO, the *Ktedonobacteraceae* included in this study carry a Form I RuBisCO. The presence of *rbcLS* genes in members of the *Ktedonobacteraceae* from silica-rich oligotrophic caves indicates that the TAL variant of the CBB cycle can also occur in association with the Form I RuBisCO, expanding the current knowledge of CBB cycle variants. This is particularly important considering that other bacterial isolates of the same family, including members of the genus *Thermogemmatispora*, possess genes associated with atmospheric gas utilization but do not have genes encoding enzymes involved in CO₂ fixation [62]. These findings suggest that, in *Ktedonobacteria*, the genetic traits associated with the capacity to utilize atmospheric trace gases are evolutionarily conserved among different lineages and largely independent of habitat. In contrast, the chemolithoautotrophic potential linked to the non-canonical CBB cycle may have been acquired via HGT. This is suggested by the presence of the same novel Form I subtype of RuBisCO (here proposed as IG) in *Ktedonobacteria* as well as in thermoacidophilic facultative chemolithoautotrophic members of different phyla that share the same ecological niches.

Recently, six novel MAGs of Ktedonobacteria have been reported from acidic samples collected in a basaltic lava tube (Tunnel Cave) characterized by a high organic carbon content (TOC = 9–31% w/w) [6]. These Ktedonobacteria lacked RuBisCO genes, possibly reflecting the higher availability of organic substrates, the cave’s shallow depth (approximately 100 m in length), and the nature of the sampled material (organic-rich floor deposits). Together, these features indicate a stronger influence of external inputs compared to the extreme oligotrophy of the silica-rich caves analysed in this study (organic carbon in Imawarì Yeutà Cave and Warren Cave was <0.1% w/w). These results suggest that the oligotrophic conditions of subterranean environments may have acted as selective pressures favouring the occurrence of RuBisCO genes and associated traits for dark CO₂ fixation in Ktedonobacteria.

While the occurrence of genes associated with chemolithoautotrophic metabolisms within the Ktedonobacteria class is a novel finding, the carbon fixation supported by the oxidation of atmospheric trace gases has previously been reported in other oligotrophic environments such as desert soils from various climatic regions [61, 63]. This process, known as atmospheric chemosynthesis or more recently defined as aerotrophy, has been proposed as a major mechanism supporting chemoautotrophic primary production in desert ecosystems where photosynthetic input is low and organic compounds are scarce [64]. However, unlike desert soils, where both aerotrophy and, to a lesser extent, photosynthesis contribute to primary production [63], deep and oligotrophic caves lack photosynthetic activity. In these ecosystems, we found that Ktedonobacteria genomes carry functional traits associated with the capacity to use atmospheric trace gases and sporadic organic carbon inputs as alternative energy sources to light. However, it should be acknowledged that these observations are based on a limited number of silica-rich oligotrophic cave systems; moreover, further studies integrating targeted isolation strategies with atmospheric trace gas consumption would be required to assess the inferred metabolic activities and the role of Ktedonobacteria in supporting primary production. Despite these limitations, our findings provide a genomic framework suggesting how Ktedonobacteria may contribute to sustaining primary production through aerotrophy, potentially supporting the establishment of the rich and diverse microbial communities that characterize silica-rich oligotrophic caves.

## Supplementary Material

Supplementary_material_ycag175

## Data Availability

Illumina and Oxford Nanopore Technologies (ONT) sequencing reads from samples Ay302 and Ay304 are available in the NCBI under BioProject accession PRJNA610757. Additional datasets deposited in Figshare (DOI: 10.6084/m9.figshare.30657227) are (i) genomes and MAGs included in the pangenome analysis; (ii) raw data and R scripts used to reproduce Figs 3, 4, and  7 as well as the PERMANOVA analyses; (iii) anvi’o genome and pangenome databases, including the pangenome summary file and pangenome enrichment results; (iv) IQTREE input and output files used for the *rbcL* phylogenetic reconstruction (Fig. 5); (v) input and output files from the hydrogenase classification analysis via HydDB; and (vi) input and intermediate files from the targeted SBPase and bifunctional FBPase/SBPase search via tBLASTN and MMseq2.
