# Supplementary material for: Genomic evidence for aerotrophy as a defining trait of Ktedonobacteria inhabiting silica-rich oligotrophic caves
Source: ISME Commun. 2026 Jun 22;6(1):ycag175. doi: 10.1093/ismeco/ycag175 (PMC13374853; doi:10.1093/ismeco/ycag175)
Supplement: Supplementary_material_ycag175 [file supplementary_material_ycag175.zip › table_s2_rev_ycag175_CORRECT.docx]

**Table S2**. Description of the silica-rich oligotrophic cave systems and corresponding samples included in this study

| **Cave** | **Sample name** | **Location and geological substrate** | **Description** | **Mineralogy** | **SiO_2_ (%)** | **Organic carbon (%)** | **pH** | **Temperature**  **(° C)** | **Reference(s)** |
| --- | --- | --- | --- | --- | --- | --- | --- | --- | --- |
| Imawarì Yeutà | Ay304 | Auyàn Tepui, Venezuela; quartz-sandstone | White dendritic biofilm on a quartzite rock on the cave floor | Quartz (SiO_2_) | >98 | 0.095 ± 0.009^a^ | 5 | 14.9 | [12, 13] |
| Imawarì Yeutà | Ay317 | Auyàn Tepui, Venezuela; quartz-sandstone | Pristine quartzite cave wall | Quartz (SiO_2_) | >98 | 0.063 ± 0.010^a^ | 4 | 14.9 | [9, 12] |
| Imawarì Yeutà | Ay302 | Auyàn Tepui, Venezuela; quartz-sandstone | White part of the amorphous silica speleothem on a quartzite rock wall | Quartz, Opal-G (SiO_2_) | >98 | 0.070 ± 0.024^a^ | 5 | 14.9 | [9, 12] |
| Warren | - | Mt Erebus, Antarctica; Fumarolic ice cave formed in volcanic rocks | Sandy sediment | Anorthoclase feldspar [(Na,K)AlSi_3_O_8_)] | 56 | 0.0126^b^ | 5.2 | 14.6 | [18] |
| Monte Cristo | P7 | Espinhaço Mountains in San Francisco craton, northern Brazil; Proterozoic metamorphic quartzite | Wet brownish saprolite (weathered rock) | Quartz (SiO_2_), muscovite  [KAl_2_(Si_3_Al)O_10_(OH, F)_2_],  kaolinite [Al_2_Si_2_O_5_(OH)_4_],  rutile (TiO_2_) | >90^c^ | NR^d^ | 5.5-7.1 | ~20.0^e^ | [17] |

^a^ Organic carbon measured in this study on quartzite substrate adjacent to the microbial sample.

^b^ Organic carbon reported by Tebo et al. (2015)

^c^ Typical % of SiO_2_ in a quartzite cave

^d^ Not reported in the original study

^e^ Mean annual temperature of Diamantina, Minas Gerais (Brazil)
